# Supplementary material for: Short-term action-effect bindings encode perceptual transitions rather than perceptual end-states
Source: Psychon Bull Rev. 2026 Jul 15;33(6):190. doi: 10.3758/s13423-026-02966-9 (PMC13372912; doi:10.3758/s13423-026-02966-9)
Supplement: Supplementary file 1 — Supplementary file1 (DOCX 2196 KB) [file 13423_2026_2966_MOESM1_ESM.docx]

# Supplementary Material A: Experiments 2 and 3

## Experiment 2

A minor limitation of Experiment 1 is that the probe type was manipulated within participants. This design choice not only halved the number of trials for the diagnostic probe offset condition, potentially lowering statistical power (Miller, 2024), but also introduced the possibility of contrast effects from the condition with onset probes. Experiment 2 addresses this issue by exclusively using offset probes.

### Participants

One-hundred twenty new participants (47 females; *M*_age_=40.9 years, *SD*=12.5) were recruited online. Five additional participants were replaced due to high error rates (>1/3).

### Method

The method was as in Experiment 1, but we included only the probe offset condition and removed the probe onset condition. Further, participants were not discouraged from always repeating the prime response. As in Experiment 1, they completed four blocks of 48 trials each.

### Results

We removed catch trials (33.3%) and trials with errors (6.1%). The rate of response repetitions was analyzed using a paired *t*-test that compared end-state repetitions versus end-state alternations. We again observed no action-effect binding effect (73.4% vs. 73.7%), *t*(119)=-0.15, *p*=.883, *d_z_*=-0.01, BF_01_=9.76.

### Discussion

Experiment 2 was a replication of the probe offset condition because it is diagnostic for the involvement of change-based coding. Despite our high statistical power, we again observed no action-effect binding effect when the same end-states were achieved through different transitions. Therefore, this null finding cannot be explained by a potential contrast with onset probes, because participants were not exposed to such onset probes at all.

Moreover, Experiment 2 revealed an overall bias towards above-chance response repetitions that was more pronounced than in Experiment 1, most likely due to slight change in the instructions. Deviations from chance are common in action-effect binding paradigms, with repetition rates reported below chance (Dutzi & Hommel, 2009, Exp. 1; Janczyk et al., 2023, Exp. 3), at chance (Dutzi & Hommel, 2009, Exp. 2, 3, and 4; Moeller et al., 2016), and above chance (Foerster et al., 2022; Foerster, Linz, et al., 2026; Mocke et al., 2026, Exp. 2). One possible post-hoc account could be that task demands modulate the relative weighting of competing sequential tendencies. On the one hand, sequential behavior may exhibit an explicit alternation bias (e.g., the so-called *Gambler’s Fallacy*; Tversky & Kahneman, 1971; see also Laplace, 1814/1902), reducing repetition rates when random responding is strongly emphasized, for example with un-speeded random actions in the prime phase. On the other hand, sequential behavior may exhibit positive recency effects (e.g., a facilitation of recently executed responses; Bertelson, 1961; Foerster, Schaaf, et al., 2026; Foerster, Moeller, et al., 2026), increasing repetition rates when random responding is only weakly emphasized or a secondary task demand, for example with speeded classification actions in the prime phase (cf. Janczyk et al., 2012, Exp. 1). Although highly speculative, this account is consistent with evidence that behavior in sequential tasks can track recent events even if this conflicts with explicit judgements of randomness (Perruchet, 1985). Therefore, future work should integrate action-effect binding paradigms with established theories and approaches to random sequence generation (e.g., Baddeley, 1966; Bar-Hillel & Wagenaar, 1991; Guseva et al., 2023; Naefgen & Janczyk, 2018).

## Experiment 3

As another alternative explanation for the results of Experiment 1, action-effect associations (Rescorla, 1991) as well as binding and retrieval might be context-dependent (e.g., Frings et al., 2017; Mayr et al., 2018; Münster & Frings, 2025; Moeller et al., 2025; Qiu et al., 2023). Thus, the absent action-effect binding effect for the probe offset condition may have resulted from the different start-states in prime and probe, which may have been interpreted as a context switch. To test for such context-dependency, Experiment 3 introduced a strong visual context switch in both probe types.

### Participants

One-hundred twenty new participants (60 females; *M*_age_=39.0 years, *SD*=12.6) were recruited online. Nine additional participants were excluded due to high error rates (>1/3).

### Method

The method was the same as in Experiment 1, but we changed the probe’s color scheme so that it constituted a salient context switch relative to the unchanged prime display: In Experiment 3, the probe background was dark grey instead of black, and the rectangles of the probe stimulus were black instead of dark grey (see Fig. S1).

### Results

We removed catch trials (33.3%) and trials with errors (6.9%). Data were analyzed as in Experiment 1 (see Fig. S1).

There were more response repetitions when the end-state repeated rather than alternated (67.5% vs. 64.0%), *F*(1,119)=6.47, *p*=.012, $\eta_{p}^{2}$=.05. There was no significant main effect of probe type, *F*(1,119)=2.44, *p*=.121, $\eta_{p}^{2}$=.02, BF_01_=3.97. Crucially, end-state relation and probe type interacted, *F*(1,119)=26.23, *p*<.001, $\eta_{p}^{2}$=.18, with a highly significant action-effect binding effect for onset probes (69.8% vs. 59.5%), *t*(119)=4.89, *p*<.001, *d_z_*=0.45, and no reliable action-effect binding effect for offset probes (65.3% vs. 68.5%), *t*(119)=-1.88, *p*=.063, *d_z_*=-0.17, BF_01_=1.81 (descriptively opposite to what would be expected under binding and retrieval of end-states; non-preregistered, directional BF_0+_=27.4).


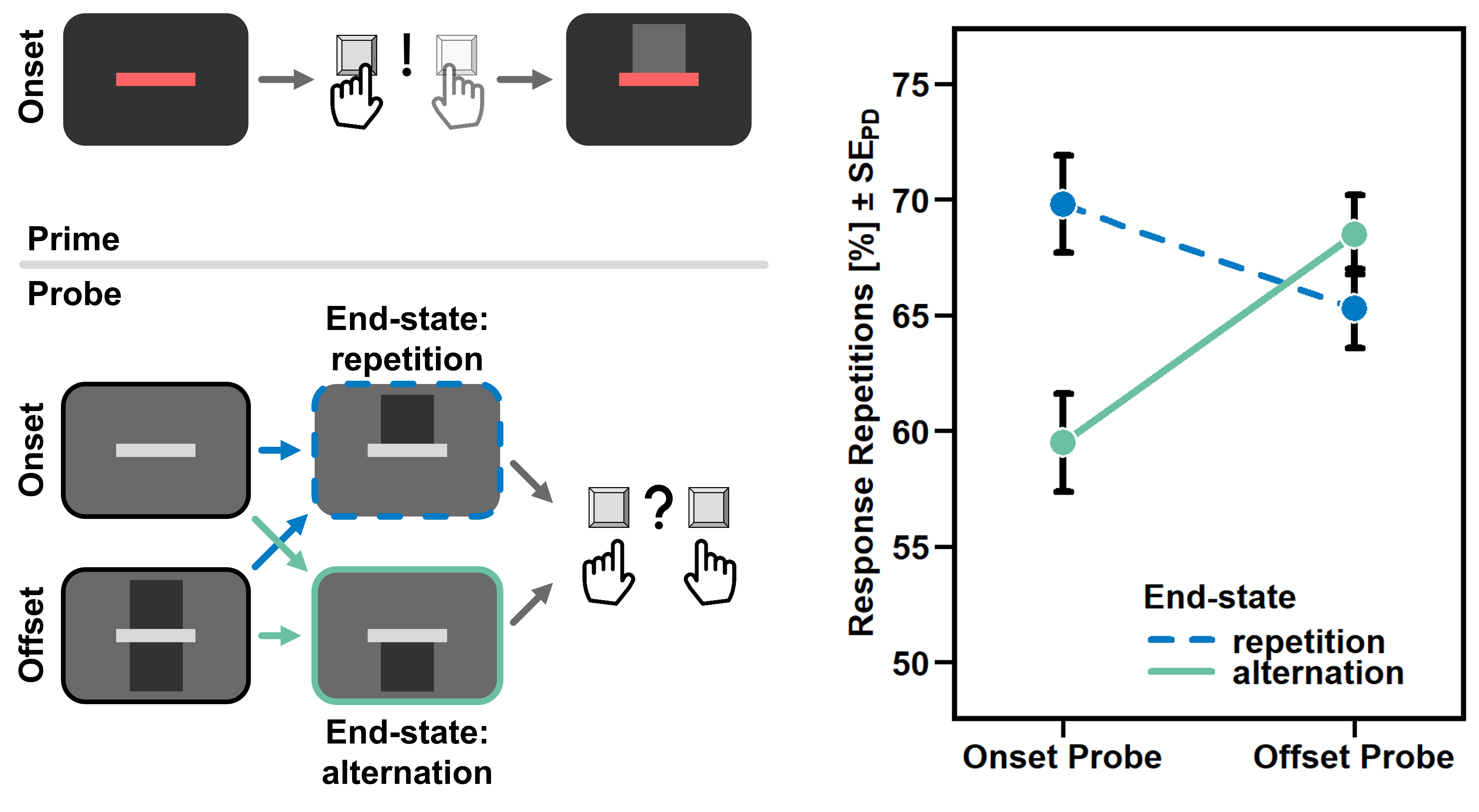


**Figure S1. Design and Results of Experiment 3.** Experiment 3 was exactly as Experiment 1, but for the probe, the colors of the background and of the stimulus rectangles were swapped. Again, the rate of response repetitions revealed reliable action-effect binding only if the end-state was achieved by the same transition, but not if it was achieved by a different transition. Error bars denote the standard error of paired differences, computed separately for the onset and offset condition (Pfister & Janczyk, 2013).

### Exploratory Between-Experiments Analysis

Based on a reviewer suggestion, we additionally conducted a between-experiments comparison to assess whether the context switch from prime to probe modulates the action-effect binding effect. Therefore, we conducted a 2 × 2 × 2 ANOVA with end-state relation (repetition vs. alternation) and probe type (onset vs. offset) as within-subjects factors and experiment (Experiment 1 vs. Experiment 3) as between-subjects factor. None of the effects involving experiment were significant. Specifically, there was no main effect of experiment, *F*(1,238)=0.43, *p*=.511, $\eta_{p}^{2}$<.01, no interaction of experiment and end-state relation, *F*(1,238)=1.98, *p*=.161, $\eta_{p}^{2}$=.01, no interaction of experiment and probe type, *F*(1,238)=1.98, *p*=.160, $\eta_{p}^{2}$=.01, and no three-way interaction, *F*(1,238)=0.53, *p*=.469, $\eta_{p}^{2}$<.01.

Further, we also focused only on trials with onset probes and conducted a corresponding 2 × 2 ANOVA with end-state relation (repetition vs. alternation) as within-subjects factor and experiment (Experiment 1 vs. Experiment 3) as between-subjects factor. Again, no effects involving experiment were observed: Neither the main effect of experiment, *F*(1,238)=0.02, *p*=.888, $\eta_{p}^{2}$<.01, nor the interaction, *F*(1,238)=2.21, *p*=.138, $\eta_{p}^{2}$=.01, were significant.

### Discussion

To test whether a context switch abolishes action-effect binding, Experiment 3 conceptually replicated Experiment 1 and introduced a color change from prime to probe (similarly for both onset and offset probes). As in Experiment 1, a robust action-effect binding effect was observed for onset probes, and exploratory between-experiment comparisons indicated that this effect was not modulated by the color manipulation. While this finding is consistent with prior evidence that task-irrelevant pre-response perceptual features do not influence action-effect bindings (Moeller et al., 2019), it does not exclude the possibility that an even stronger context manipulation may do so. In particular, onset and offset probes differ in the spatial interpretation of their start displays, which may be a more effective context than a color inversion. Accordingly, it remains possible that the absence of action-effect binding effects in offset probes reflects differences in start-states rather than transitions.

# Supplementary Material B: Mixed-Effects Model Analysis

Based on a reviewer suggestion, we additionally analyzed the rate of response repetitions from prime to probe using a generalized linear mixed-effects model (GLMM) with a binomial distribution and logit link. For Experiment 1, we used the *lme4* package v1.1.38 (Bates et al., 2015) to fit response repetitions as a function of end-state relation (repetition vs. alternation) and probe type (onset vs. offset), including their interaction, with participants as random intercepts. End-state relation was effect-coded with repetition as 0.5 and alternation as -0.5, and probe type was effect-coded with onset as 0.5 and offset as -0.5. Follow-up comparisons were conducted using the *emmeans* package v2.0.1 (Lenth & Piaskowski, 2025). In addition to the frequentist model, we used the *brms* package v2.23.0 (Bürkner, 2021) to fit a Bayesian mixed-effects model with the same fixed- and random-effects structure, a Bernoulli likelihood with logit link, and default priors.

For Experiment 1, the GLMM revealed a significant main effect of end-state relation, *z*=9.72, *p*<.001, but no significant main effect of probe type, *z*=1.39, *p*=.165. Crucially, end-state relation and probe type interacted, *z*=11.37, *p*<.001, with a highly significant action-effect binding effect for onset probes, odds ratio (OR)=2.36, *z*=14.78, *p*<.001, and no action-effect binding effect for offset probes, OR=0.94, *z*=‑1.18, *p*=.237.

The Bayesian mixed-effects model showed a credible main effect of end-state relation, β=0.40, 95% credible interval (CI)=[0.32, 0.48], but no credible main effect of probe type, β=0.06, 95% CI=[‑0.03, 0.14]. Crucially, the interaction was also credible, β=0.93, 95% CI=[0.77, 1.09], with a strong action-effect binding effect for onset probes, OR=2.36, 95% CI=[2.10, 2.64], and no credible action-effect binding effect for offset probes, OR=0.94, 95% CI=[0.83, 1.04].

For Experiment 2, data were analyzed as in Experiment 1, but because only offset probes were used, the factor probe type and the interaction were omitted, reducing both models to the single predictor end-state relation.

The GLMM revealed no significant action-effect binding effect, OR=0.98, *z*=‑0.41, *p*=.683. Likewise, the Bayesian mixed-effects model showed no credible action-effect binding effect, OR=0.98, 95% CI=[0.90, 1.07].

For Experiment 3, data were analyzed as in Experiment 1. The GLMM revealed a significant main effect of end-state relation, *z*=5.03, *p*<.001, and a significant main effect of probe type, *z*=‑2.56, *p*=.010. Crucially, end-state relation and probe type interacted, *z*=9.58, *p*<.001, with a highly significant action-effect binding effect for onset probes, OR=1.81, *z*=10.34, *p*<.001, and an inverse pattern for offset probes, OR=0.83, *z*=‑3.22, *p*=.001.

The Bayesian mixed-effects model showed a credible main effect of end-state relation, β=0.20, 95% CI=[0.12, 0.28], and a credible main effect of probe type, β=‑0.10, 95% CI=[‑0.18, ‑0.02]. Crucially, the interaction was also credible, β=0.78, 95% CI=[0.62, 0.93], with a strong action-effect binding effect for onset probes, OR=1.81, 95% CI=[1.61, 2.02], and an inverse pattern for offset probes, OR=0.83, 95% CI=[0.74, 0.93].

# References

Baddeley, A. D. (1966). The capacity for generating information by randomization. *The Quarterly Journal of Experimental Psychology*, *18*(2), 119–129. https://doi.org/10.1080/14640746608400019

Bar-Hillel, M., & Wagenaar, W. A. (1991). The perception of randomness. *Advances in Applied Mathematics*, *12*(4), 428–454. https://doi.org/10.1016/0196-8858(91)90029-I

Bates, D., Mächler, M., Bolker, B., & Walker, S. (2015). Fitting linear mixed-effects models using lme4. *Journal of Statistical Software*, *67*(1), 1–48. https://doi.org/10.18637/jss.v067.i01

Bertelson, P. (1961). Sequential redundancy and speed in a serial two-choice responding task. *The Quarterly Journal of Experimental Psychology*, *13*(2), 90–102. https://doi.org/10.1080/17470216108416478

Bürkner, P.‑C. (2021). Bayesian item response modeling in R with brms and stan. *Journal of Statistical Software*, *100*(5), 1–54. https://doi.org/10.18637/jss.v100.i05

Dutzi, I. B., & Hommel, B. (2009). The microgenesis of action-effect binding. *Psychological Research*, *73*(3), 425–435. https://doi.org/10.1007/s00426-008-0161-7

Foerster, A., Linz, S., Moeller, B., Nemeth, M., Frings, C., & Pfister, R. (2026). Feature binding and error commission. *Attention, Perception & Psychophysics*, *88*, Article 50. https://doi.org/10.3758/s13414-025-03164-w

Foerster, A., Moeller, B., Huffman, G., Kunde, W., Frings, C., & Pfister, R. (2022). The human cognitive system corrects traces of error commission on the fly. *Journal of Experimental Psychology: General*, *151*(6), 1419–1432. https://doi.org/10.1037/xge0001139

Foerster, A., Moeller, B., Nemeth, M., Schaaf, M., Frings, C., & Pfister, R. (2026). Binding continuous response features of extended movements: Integration with discrete response but not stimulus features. *Psychological Research*, *90*, Article 77. https://doi.org/10.1007/s00426-026-02295-5

Foerster, A., Schaaf, M., Weissman, D. H., Kunde, W., & Pfister, R. (2026). Response activation in error processing: Assessing leakage into upcoming action episodes. *Journal of Experimental Psychology: Human Perception and Performance*, *52*(4), 516–538. https://doi.org/10.1037/xhp0001402

Frings, C., Koch, I., & Moeller, B. (2017). How the mind shapes action: Offline contexts modulate involuntary episodic retrieval. *Attention, Perception & Psychophysics*, *79*(8), 2449–2459. https://doi.org/10.3758/s13414-017-1406-6

Guseva, M., Bogler, C., Allefeld, C., & Haynes, J.‑D. (2023). Instruction effects on randomness in sequence generation. *Frontiers in Psychology*, *14*, Article 1113654. https://doi.org/10.3389/fpsyg.2023.1113654

Janczyk, M., Giesen, C. G., Moeller, B., Dignath, D., & Pfister, R. (2023). Perception and action as viewed from the theory of event coding: A multi-lab replication and effect size estimation of common experimental designs. *Psychological Research*, *87*(4), 1012–1042. https://doi.org/10.1007/s00426-022-01705-8

Janczyk, M., Heinemann, A., & Pfister, R. (2012). Instant attraction: Immediate action-effect bindings occur for both, stimulus- and goal-driven actions. *Frontiers in Psychology*, *3*, Article 446. https://doi.org/10.3389/fpsyg.2012.00446

Laplace, P. S. (1902). *A philosophical essay on probabilities* (F. W. Truscott & F. L. Emory, Trans.). John Wiley & Sons. (Original work published 1814)

Lenth, R. V., & Piaskowski, J. (2025). *emmeans: Estimated Marginal Means, aka Least-Squares Means*. https://CRAN.R-project.org/package=emmeans

Mayr, S., Möller, M., & Buchner, A. (2018). Contextual modulation of prime response retrieval processes: Evidence from auditory negative priming. *Attention, Perception & Psychophysics*, *80*(8), 1918–1931. https://doi.org/10.3758/s13414-018-1574-z

Miller, J. (2024). How many participants? How many trials? Maximizing the power of reaction time studies. *Behavior Research Methods*, *56*(3), 2398–2421. https://doi.org/10.3758/s13428-023-02155-9

Mocke, V., Kunde, W., & Schreiner, M. R. (2026). Compatible effects enhance short-term action-effect binding. *Journal of Experimental Psychology: Learning, Memory, and Cognition*, *52*(4), 522–532. https://doi.org/10.1037/xlm0001503

Moeller, B., Beste, C., Münchau, A., & Frings, C. (2025). Large scale event segmentation affects the microlevel action control processes. *Journal of Experimental Psychology: General*, *154*(4), 969–979. https://doi.org/10.1037/xge0001681

Moeller, B., Pfister, R., Kunde, W., & Frings, C. (2016). A common mechanism behind distractor-response and response-effect binding? *Attention, Perception & Psychophysics*, *78*(4), 1074–1086. https://doi.org/10.3758/s13414-016-1063-1

Moeller, B., Pfister, R., Kunde, W., & Frings, C. (2019). Selective binding of stimulus, response, and effect features. *Psychonomic Bulletin & Review*, *26*(5), 1627–1632. https://doi.org/10.3758/s13423-019-01646-1

Münster, N. D., & Frings, C. (2025). Red and green and the mind in between: How context modulates feature relations in action-perception integration. *Journal of Experimental Psychology: Human Perception and Performance*, *51*(11), 1502–1512. https://doi.org/10.1037/xhp0001359

Naefgen, C., & Janczyk, M. (2018). Free choice tasks as random generation tasks: An investigation through working memory manipulations. *Experimental Brain Research*, *236*(8), 2263–2275. https://doi.org/10.1007/s00221-018-5295-2

Perruchet, P. (1985). A pitfall for the expectancy theory of human eyelid conditioning. *The Pavlovian Journal of Biological Science*, *20*(4), 163–170. https://doi.org/10.1007/BF03003653

Pfister, R., & Janczyk, M. (2013). Confidence intervals for two sample means: Calculation, interpretation, and a few simple rules. *Advances in Cognitive Psychology*, *9*(2), 74–80. https://doi.org/10.5709/acp-0133-x

Qiu, R., Möller, M., Koch, I., Frings, C., & Mayr, S. (2023). The influence of event segmentation by context on stimulus-response binding. *Journal of Experimental Psychology: Human Perception and Performance*, *49*(3), 355–369. https://doi.org/10.1037/xhp0001093

Rescorla, R. A. (1991). Associative relations in instrumental learning: The eighteenth Bartlett memorial lecture. *The Quarterly Journal of Experimental Psychology Section B*, *43*(1), 1–23. https://doi.org/10.1080/14640749108401256

Tversky, A., & Kahneman, D. (1971). Belief in the law of small numbers. *Psychological Bulletin*, *76*(2), 105–110. https://doi.org/10.1037/h0031322
